# Facial Paralysis Detection in Infrared Thermal Images Using Asymmetry Analysis of Temperature and Texture Features

**DOI:** 10.3390/diagnostics11122309

**Published:** 2021-12-08

**Authors:** Xulong Liu, Yanli Wang, Jingmin Luan

**Affiliations:** Department of Biomedical Engineering, School of Computer and Communication Engineering, Northeastern University, Qinhuangdao 066004, China; yanliwang@stumail.neu.edu.cn (Y.W.); luanjingmin@neuq.edu.cn (J.L.)

**Keywords:** facial paralysis, infrared thermal images, thermal asymmetry, temperature features, texture features

## Abstract

Facial temperature distribution in healthy people shows contralateral symmetry, which is generally disrupted by facial paralysis. This study aims to develop a quantitative thermal asymmetry analysis method for early diagnosis of facial paralysis in infrared thermal images. First, to improve the reliability of thermal image analysis, the facial regions of interest (ROIs) were segmented using corner and edge detection. A new temperature feature was then defined using the maximum and minimum temperature, and it was combined with the texture feature to represent temperature distribution of facial ROIs. Finally, Minkowski distance was used to measure feature symmetry of bilateral ROIs. The feature symmetry vectors were input into support vector machine to evaluate the degree of facial thermal symmetry. The results showed that there were significant differences in thermal symmetry between patients with facial paralysis and healthy people. The accuracy of the proposed method for early diagnosis of facial paralysis was 0.933, and the area under the ROC curve was 0.947. In conclusion, temperature and texture features can effectively quantify thermal asymmetry caused by facial paralysis, and the application of machine learning in early detection of facial paralysis in thermal images is feasible.

## 1. Introduction

Peripheral facial paralysis is caused by dysfunction of the facial nerve, which is characterized by imbalance of facial expression muscles on one side [1]. The most common peripheral facial paralysis is idiopathic facial paralysis (Bell’s palsy) [2]. Most patients with facial paralysis have a good prognosis; however, around 15% of patients cannot fully recover; instead, they will have sequelae, such as oblique eyes and crooked mouth [3,4]. If facial paralysis cannot be diagnosed and treated early, it may lead to poor recovery of the facial nerve, affecting patients’ appearance as well as their quality of life [5].

Facial paralysis is mainly diagnosed by physicians to evaluate the facial nerve function of patients according to their facial symptoms using the facial nerve grading systems, such as House-Brackmann score (HB score) [6,7,8]. Patients are asked to perform a series of movements such as frowning, closing eyes, smiling, and puffing cheeks, to determine the symmetry of the patient’s facial expression between ipsilateral and contralateral facial regions, and assess the severity of facial paralysis using the degree of symmetry [9]. The reliability of the HB score depends on the physician’s subjective experience and the patient’s cooperation [10]. When the initial symptoms of facial paralysis are not obvious, the reliability of the HB score is low [11]. Electromyography (EMG) can objectively diagnose facial paralysis by checking the conduction velocity of the facial nerve to determine the degree of facial nerve damage [2]. However, EMG has low sensitivity in detecting mild to moderate facial nerve palsy in the acute phase of facial paralysis. It is mainly used for the prognosis of facial paralysis and is not suitable for its early diagnosis. In addition, blink reflex is currently one of the most sensitive electrophysiological indicators for early diagnosis of facial paralysis [11]. However, it requires a series of stimuli to induce the defensive reflex of the subject; electrode pads are attached to specific areas of the face causing pain and discomfort to the subject, which results in poor coordination on the part of the subject. Therefore, the development of an objective, quantitative, and easy-to-use early diagnosis method for facial paralysis can be a useful supplement to the HB score and electrophysiological examination.

Facial paralysis is associated with local vasospasm and tissue edema caused by dysfunction, ischemia, or inflammation of the facial nerve and surrounding tissues [11]. Therefore, the onset of facial paralysis will affect the local blood circulation of the patient’s face and change the normal temperature distribution [12]. For a healthy person, the temperature distribution on the face is symmetrical [13]. Previous studies [14] show that there is a difference in the symmetry of facial temperature distribution between facial paralysis patients and healthy people. Further, facial paralysis can be diagnosed early by measuring the degree of facial thermal asymmetry in patients.

Infrared thermal imaging (IRT) is a noncontact, nonradiation, and easy-to-use temperature measurement tool [15]. It involves temperature measurement and thermal asymmetry analysis of the facial regions of interest (ROIs) and has been widely used for diagnosis and assessment of some facial diseases [16,17,18,19], such as dry eye [20], temporomandibular disorders [21,22], chronic migraine [23], and facial paralysis [14,24,25]. Existing studies [26,27] used IRT to measure the temperature difference between the left and right sides of the face of facial paralysis patients; this difference was related to the severity of facial paralysis [28,29]. The temperature difference in facial paralysis patients was significantly greater than that in healthy people [12]. Moreover, the feasibility of IRT assisted early diagnosis of facial paralysis was evaluated [14]; the results showed that facial paralysis on thermal images can be preliminarily detected by thermal asymmetry analysis; however, more effective temperature distribution representation is required. Texture features are used to represent the spatial distribution of temperature on thermal images, and they have been used in the detection of diabetic foot ulcers [30] and breast cancer [31,32,33]. Liu et al. [34] used texture features to evaluate the thermal asymmetry of facial paralysis, and preliminarily validated their feasibility in the diagnosis of facial paralysis. However, the above studies have three limitations: (1) they only focus on analyzing temperature or texture features; (2) the ROI segmentation is manual, which reduces the reliability of thermal image analysis; and (3) without using machine learning, threshold segmentation cannot obtain optimal results.

To resolve the shortcomings of the aforementioned studies, this study aims to develop a computer-assisted facial paralysis early diagnosis system using IRT through combining temperature and texture features. Automatic segmentation of ROIs was adopted to improve the reliability of thermal image analysis. A new temperature feature was proposed by combining maximum and minimum temperature values. Texture features were introduced to represent the spatial distribution of temperature. Further, the Minkowski distance was used to measure the symmetry between the ipsilateral and contralateral features of facial ROIs. Support vector machine (SVM) was used to evaluate the difference in facial thermal symmetry between facial paralysis patients and healthy people, so as to realize the early detection of facial paralysis in thermal images.

## 2. Materials and Methods

**Figure 1:**
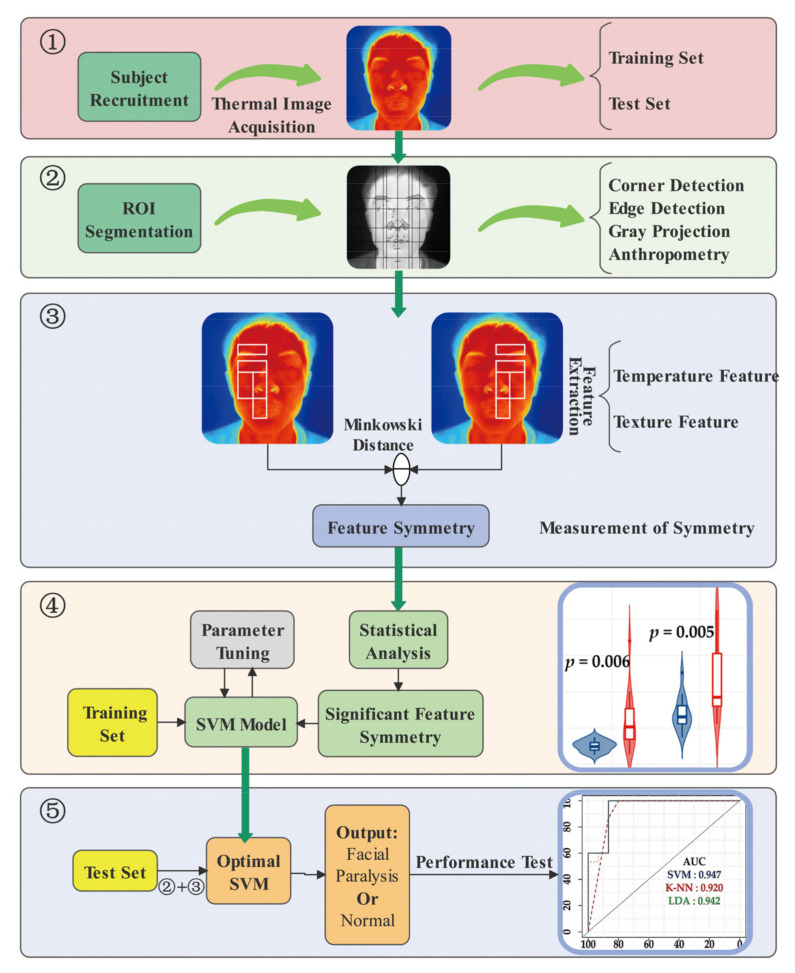
Overview of proposed classification method for early detection of facial paralysis.

This paper proposes a computer-assisted thermographic analysis method for early detection of facial paralysis. The general process is shown in Figure 1. The whole method is divided into five stages. The first stage is the recruitment of subjects and the collection of thermal images. The collected thermal images are divided into training and test datasets. The purpose of the training dataset is to construct an optimal classification model using the principle of minimizing the training error. The test dataset is used to evaluate the generalization performance of the classifier. The second stage is the automatic segmentation of the facial ROIs. Using corner and edge detection combined with anthropometry, the subject’s facial thermal image is divided into 10 symmetrical ROIs. The third stage is feature extraction and symmetry measurement. The temperature and texture features of each ROI are extracted separately, and Minkowski distance is used to measure the feature symmetry, which refers to the similarity between the features of the two ROIs on the left and right sides. The fourth stage is statistical analysis and feature selection. The t-test is used to determine the feature symmetry, which has significant differences between the facial paralysis patients and the healthy population. The feature symmetry is used as the classifier input, and the optimal parameters of the classifier are calculated using the training dataset. The fifth stage uses the test dataset to measure the generalization performance of the proposed method for the diagnosis of facial paralysis.

### 2.1. Subjects

A total of 90 subjects were involved in this study, including 45 patients with facial paralysis and 45 healthy people. The healthy subjects consisted of 24 males and 21 females with average age and body mass index of 41.20 ± 9.02 years and 23.85 ± 2.31 kg/m^2^, respectively. Patients with facial paralysis consisted of 22 males and 23 females with average age of 44.37 ± 8.14 years and body mass index of 23.12 ± 2.36 kg/m^2^, respectively. The Ethics Committee of Northeastern University approved the study. All volunteers were recruited from facial paralysis patients at Qinhuangdao Hospital of Traditional Chinese Medicine and healthy workers at Northeastern University at Qinhuangdao. The subjects were fully informed of the experimental procedures and precautions before enrollment, and they signed the informed consent form. The inclusion criteria of patients with facial paralysis were: (1) unilateral Bell’s palsy, (2) having symptoms that appeared at most more than 72 h prior, and (3) taking no medication or acupuncture treatment. The exclusion criteria for all subjects were: (1) age less than 18 years, (2) chronic diseases such as hypertension, diabetes, and gout, (3) suffering from migraine, rhinitis, conjunctivitis, dry eye, temporomandibular arthritis, gingivitis and other diseases that may change the normal temperature distribution of the face, and (4) cosmetic surgery.

All subjects were evaluated by the experienced clinicians based on the HB score. The purpose of this study is to preliminarily verify the feasibility of applying infrared thermography and machine learning in the early diagnosis of facial paralysis. Therefore, all subjects were divided into two groups (normal and facial paralysis), and the facial nerve function was graded as shown in Table 1. In the table, the numbers of the patients with different HB scores is non-uniform distribution, which is consistent with the characteristics of disease onset. The numbers of the patients with grade IV and V are much more than that with grade II and III.

### 2.2. Acquisition of Thermal Images

The thermal camera used for this study was the FLIR Tau 336 (FLIR Systems, Inc. Wilsonville, OR, USA), with a resolution of 336 × 256 pixels and a thermal sensitivity of 0.05 °C, which works for an emissivity value of 0.98 [13]. The thermal image acquisition was carried out in an indoor environment, where the indoor temperature was controlled at 24–25 °C and the humidity was maintained at 45–55%. Before the acquisition, all subjects were screened through questionnaires to exclude those who did not meet the thermal image acquisition criteria [35], including those who smoked, drunk alcoholic or caffeine-containing drinks, applied foundation cream, or had performed physical activity, within 4 h before thermal image collection. Additionally, subjects were required to rest for at least 15 min to adapt to room temperature, and the thermal imager was turned on for 15 min to stabilize the internal microbolometer. The thermal camera was placed 1.2 m away from the subjects, perpendicular to the subject’s face, and it took the front facial images of the subjects.

### 2.3. Automated Selection of ROIs

**Figure 2:**
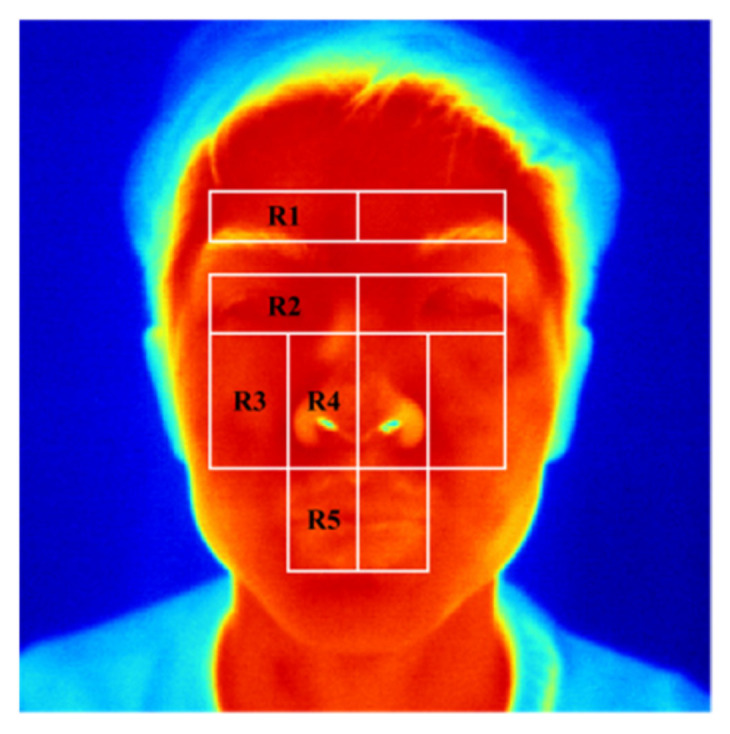
Illustration of facial ROIs. R1: forehead region; R2: orbital region; R3: infraorbital region; R4: nasal region, R5: mouth region.

**Figure 3:**
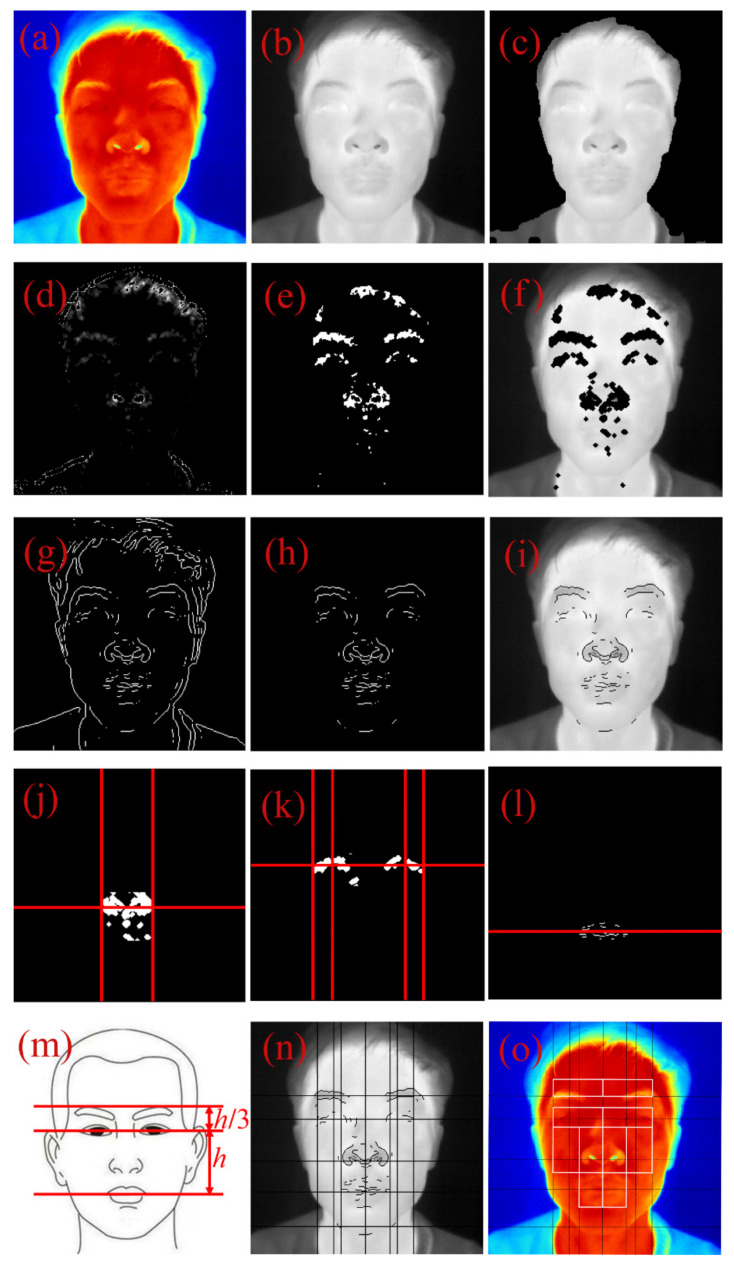
General process of automatic segmentation of facial ROIs. (**a**) Raw thermal image; (**b**) Gray processing; (**c**) Face segmentation; (**d**) Corner detection; (**e**) Image Erosion and Dilation; (**f**) Corner features; (**g**) Edge detection; (**h**) Image Erosion; (**i**) Edge features; (**j**) Gray projection of the nose; (**k**) Gray projection of the eye; (**l**) Gray projection of the mouth; (**m**) Anthropometry; (**n**) Eyebrow location; (**o**) ROI segmentation.

Based on previous studies [14] and the symptoms of facial paralysis, this study divided facial thermal images into 10 ROIs that were distributed in pairs on the left and right sides on the face, as shown in Figure 2. Automatic segmentation of facial ROIs is helpful to improve the reliability of thermal asymmetry analysis [36]. Some studies [37] used edge detection, active appearance model (AAM), or cascaded shape regression (CSR) to automatically locate key points on facial thermal images. Notably, CSR has better robustness [38]. Considering the single background and constant ambient temperature of facial thermal images in this study, an automatic segmentation algorithm of facial ROIs with low computational cost, high real-time performance was developed. Interpretability was proposed through combining corner detection, edge detection, and gray projection. The algorithm steps are shown in Table 2 and Figure 3.

The temperature in most areas of the face changes smoothly and has strong spatial correlation, which is mainly due to the uniform distribution of blood vessels in these areas. However, in the organ regions, due to changes of physiological structure, there are specific changes in temperature. For example, the surface temperature of the nostrils is closer to the ambient temperature than other parts, while the surface temperature of the eyes and mouth differ considerably from the surrounding skin temperature. These temperature changes are related to the physiological characteristics of the human face and have good generalizability. Therefore, the pixels with the largest temperature change or the maximum curvature values in the thermal image can be found through corner and edge detection, allowing the key points of facial organs to be located.

The specific processing steps are shown in Figure 3. The original temperature matrix was normalized into a grayscale map (Figure 3a,b). An optimal temperature threshold was set using the Otsu method [39] to segment the facial region from the background (Figure 3c). The Harris operator [40] was used for corner detection of the face (Figure 3d), and image erosion and dilation were performed on it (Figure 3e,f) to remove the interference of the edge of the facial contour. The horizontal and vertical coordinates of nostril and eye were determined by the gray projection method [9] (Figure 3j,k). The canny edge detector [41] was used to detect the edges of the facial region (Figure 3g–i), and gray projections and anthropometry were used to locate the corners of the mouth and the eyebrow (Figure 3l–n). Corner detection, edge detection and anthropometry were combined to fine-tune and segment the facial ROIs through these horizontal and vertical coordinates (Figure 3o).

### 2.4. Feature Extraction

Temperature features are commonly used in IRT-assisted diagnosis. Furthermore, in some abnormal physiological conditions, the spatial distribution of all temperature values in ROIs will change relative to that of healthy people. Texture features can represent the temperature distribution of the facial ROIs, which may be helpful for early diagnosis of facial paralysis [34]. Therefore, the thermal asymmetry of facial paralysis was evaluated using temperature and texture features in this study.

#### 2.4.1. Temperature Features

The mean temperature (*T_mean_*), maximum temperature (*T_max_*) [42], and minimum temperature (*T_min_*) were used to represent the temperature features, which are formulated as Equations (1)–(3), *T_mean_* is the average of all temperature values in the ROI, *T_max_* is the average of the maximum 5% of all temperature values in the ROI, and *T_min_* is the average of the minimum 5% of all temperature values in the ROI.

(1) $$T_{mean}=\frac{1}{N}\;\sum_{i=1}^{N}T_{i}$$

(2) $$T_{max}=\frac{1}{N}\;\sum_{j=N+1-\left[0.05N\right]}^{N}T_{j}$$

(3) $$T_{min}=\frac{1}{N}\sum_{j=1}^{N+1-\left[0.05N\right]}T_{j}$$

where *N* is the number of all pixels in the ROI, *T_i_* is the temperature value of each pixel in the ROI, and *T_j_* is the reordering sequence of *T_i_* in ascending order.

#### 2.4.2. Texture Features

**Figure 4:**
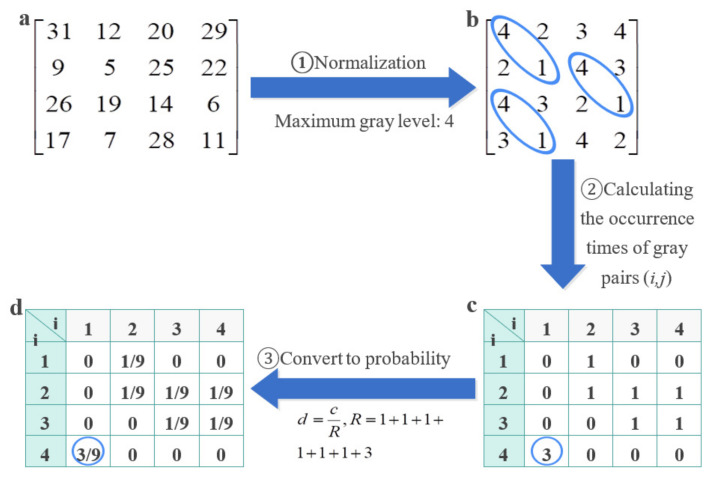
Typical GLCM calculation process. (**a**) The original thermal image is transformed into a gray matrix, and *P*(*i,j*,(1,1), 135°) is calculated. (**b**) Normalization of the original matrix to the specified gray scale range. (**c**) Counting the occurrence times of gray pair (*i*, *j*) at the two adjacent pixels on the 135° diagonal. (**d**) Calculation of the occurrence probability of each pair of pixels (*i*, *j*), namely, GLCM.

The gray level co-occurrence matrix (GLCM) is a texture feature describing the spatial distribution of temperature, in which each value represents the frequency of pixel pairs with specific value and specific spatial relationship in thermal image [30,31]. In a thermal image *I* with *M*×*N* dimensions, any pixel with coordinates (*x,y*) and another pixel with coordinates (*x + a*, *y + b*) (where *a*, *b* are integers) constitute a pixel pair. Assuming that the gray value of the pixel pair is (*i*, *j*), and the maximum gray level of the thermal image is *L*, there are a total of *L* × *L* combinations of *i* and *j*. For the whole thermal image, the number of occurrences of each (*i*, *j*) is counted, they are normalized into probability *P* (*i*, *j*) by the total number of occurrences of (*i*, *j*), and then, arranged into a square matrix, which is called GLCM. The *P*(*i*, *j* Δ*, θ*) (Equation (4)) in the GLCM is defined as follows:

(4) $$P\left(i,j,\Delta ,\theta \right)=\frac{1}{R}\left\{\begin{array}{c} 1,\;if\;I\left(x,y\right)=i\;and\;I\left(x+\Delta_{x},y+\Delta_{y}\right)=j\;\\ 0,\;Otherwise \end{array}\right.$$

where (*x, y*) is the position coordinates of the pixel in the image *I*, (*i*, *j*) is a pair of given gray values, Δ(Δ*_x_,* Δ*_y_*) is the horizontal and vertical offset between a pair of pixels, *θ* denotes the angle between the connecting line between a pair of pixels and the horizontal direction (0°, 45°, 90°, 135°), and *R* is the total number of occurrences of all possible pixel pairs in the entire image. An example of calculating GLCM is given in Figure 4.

According to previous research [14,30,31], the parameters in the GLCM in this study are as follows: the offset distance Δ is set to 2 and 5, the offset direction *θ* is set to 0°, 45°, 90°, 135°, and the maximum gray level *G* is set to 16 and 32. After calculating all 16 GLCMs and based on the given parameters, each GLCM uses 4 s-order statistics to represent the texture features of the thermal image. Table 3 lists the definitions and calculation formulas of the 4 texture features, where *μ_x_*, *σ_x_*, *μ_y_*, and *σ_y_* represent the mean and standard deviation of the rows and columns of the GLCM.

### 2.5. Symmetry Measurement

There are significant differences in the thermal symmetry of the left and right sides of the face between healthy people and facial paralysis patients [12,14]. To quantify this difference, it is necessary to measure the feature symmetry of the ROIs on the left and right sides of the face. The most commonly used representation of feature symmetry is the distance measure between two features [37,42,43]. The smaller the measured value, the better the symmetry will be.

In this study, the degree of symmetry between two temperature features is expressed as the average temperature difference (Δ*T_mean_*) and maximum temperature difference (Δ*T_max_*), which are formulated as Equations (5) and (6).

(5) $$\Delta T_{mean}=\left|T_{L\text{-}mean}-T_{R\text{-}mean}\right|$$

(6) $$\Delta T_{max}=\left\{\begin{array}{c} \Delta T_{L\text{-}max},\;if\;\Delta T_{L\text{-}max}>\Delta T_{R\text{-}max}\;\\ \Delta T_{R\text{-}max},\;Otherwise \end{array}\right.$$

where *T_L-mean_* and *T_R-mean_* represent the average temperature of the left and right ROIs respectively, and *T_L-max_* and *T_R-max_* represent the maximum temperature of the left and right ROIs, respectively. The *T_L-max_* (Equation (7)) and *T_R-max_* (Equation (8)) are defined as follows:

(7) $$\Delta T_{L\text{-}max}={\left|T_{L\text{-}max}-T_{R\text{-}min}\right|}_{\;}$$

(8) $$\Delta T_{R\text{-}max}={\left|T_{R\text{-}max}-T_{L\text{-}min}\right|}_{\;}$$

The symmetry *ρ* between two texture features is calculated by the Minkowski distance, and the *ρ* (Equation (9)) is defined as follows:

(9) $$\rho_{P,\Delta ,G}\left(F_{L,\Delta ,G},F_{R,\Delta ,G}\right)={\left(\sum_{\theta }{\left|F_{L,\Delta ,G}^{\theta }-F_{R,\Delta ,G}^{\theta }\right|}^{P}\right)}^{1/P}$$

where *F_L,Δ,G_* and *F_R,Δ,G_* represent the texture features of a pair of ROIs distributed symmetrically; *θ*, Δ, *G* are the parameters of GLCM, *θ* is the offset direction (0°, 45°, 90°, 135°), Δ is the offset distance (and the values are 2 and 5), and *G* is the maximum gray level (and the values are 16 and 32). The values of *P* are 1 and 2. When *P* = 2, it is the Euclidean distance; when *P* = 1, it is the Manhattan distance. Each ROI of the subjects has 4 types of texture features (shown in Table 2), and there are 8 types of symmetry between each type of texture features; so, there are 32 texture symmetries between each pair of ROIs of the subjects.

### 2.6. Classifier Construction

In this study, the subjects are classified as facial paralysis or normal using a support vector machine (SVM). The SVM performs binary classification of data according to supervised learning, which is suitable for small and medium-sized data samples, and non-inear, high-dimensional classification problems [44,45]. An SVM classifies samples by finding the best hyperplane. The best hyperplane refers to the hyperplane with the largest margin between two categories, and the largest margin refers to the farthest distance from all samples to the hyperplane. For a detailed introduction to the basic theory of SVM, please refer to [45].

The input of SVM is the feature symmetry vector between the ipsilateral and contralateral ROIs of the subject’s facial thermal image, and the output is the positive (facial paralysis) and the negative (normal). A subject’s facial thermal image has 5 pairs of ROIs (Figure 1), and each pair of ROIs has 2 temperature symmetries and 32 texture symmetries. Therefore, the input vector of the SVM has 170 dimensions at most. Different combinations of feature symmetries have different diagnostic values in facial paralysis. It is hypothesized that the feature symmetries with significant differences between the facial paralysis group and the normal group may have the more contribution in the diagnosis of facial paralysis. Therefore, a *t*-test (*n* = 45) was used to select these feature symmetries.

To evaluate the generalization error of different feature symmetry combinations for the diagnosis of facial paralysis, the sample set is divided into the training and test sets by the 10 repetitions of the leave-k-out cross-validation. In general, the test set should contain at least 30 samples [46]. Therefore, the value of K is set to 30. There are 90 subjects in this study, including 45 patients with facial paralysis and 45 healthy individuals. 15 patients and 15 healthy individuals are randomly selected as the test set, and the remaining 60 subjects as the training set. In the test set, at least one subject is selected in each of the HB scores from level II to level V. The results obtained by the single use of the leave-k-out method are often not reliable enough, so the leave-k-out method is repeated 10 times. The generalization performances of the classifier are the average results of the 10 tests. For example, if the *t*-test is used to select 37 out of 170 feature symmetries with significant differences, the sample set is a two-dimensional matrix with 90 rows and 37 columns. In the matrix, each row represents a subject, and each column represents a feature symmetry. The first 45 rows represent patients with facial paralysis, and the last 45 rows represent healthy people. 30 rows which are randomly selected from the matrix constitute the test set, where 15 rows are from the first 45 rows. The remaining 60 rows are used as the training set. In addition, to evaluate the effectiveness of SVM in the diagnosis of facial paralysis, two classical classifiers, i.e., k-nearest neighbor (k-NN) and linear discriminant analysis (LDA), were compared with SVM.

For the diagnosis of facial paralysis, the case can be divided into four results: true positive (TP), true negative (TN), false positive (FP) and false negative (FN) according to the combination of actual class and predicted one from the SVM. The interpretation of these classification results is shown in Table 4.

According to the confusion matrix (shown in Table 4), a total of 5 indicators were used to evaluate the diagnostic ability of the SVM classifier for facial paralysis, namely accuracy, sensitivity, specificity, precision, and F1, which are defined as follows (Equations (10)–(14)):

(10) $$\mathrm{Accuracy}\;=\;\left(\mathrm{TP}+\mathrm{TN}\right)\;÷\;\left(\mathrm{TP}+\mathrm{TN}+\mathrm{FP}+\mathrm{FN}\right)\;$$

(11) $$\mathrm{Sensitivity}=\mathrm{TP}\;÷\;\left(\mathrm{TP}+\mathrm{FN}\right)$$

(12) $$\mathrm{Specificity}=\mathrm{TN}\;÷\;\left(\mathrm{FP}+\mathrm{TN}\right)$$

(13) $$\mathrm{Precision}=\mathrm{TP}\;÷\;\left(\mathrm{FP}+\mathrm{TP}\right)$$

(14) $$F1=2\times \mathrm{Precision}\times \mathrm{Sensitivity}\;÷\;\left(\mathrm{Precision}+\mathrm{Sensitivity}\right)$$

### 2.7. Statistical Analysis

The statistical analysis was carried out with SPSS Statistics 23 (IBM, Armonk, NY, USA). The significance level was set at *p* < 0.05. The normal distributions of the temperature and texture features were verified by the Shapiro-Wilk test, and these values were expressed as Mean ± SD. The *t*-test was used to analyze the difference in the feature symmetry of facial ROIs between the facial paralysis and control group. For the feature symmetry with a statistical difference between the two groups, the inter-subject variability was evaluated by comparing the interquartile range of data in each group. The accuracy, sensitivity, specificity, precision, and F1 were used to compare the diagnostic ability of different feature symmetry combinations for facial paralysis. In addition, the area under the ROC curve (AUC) was used to compare the generalization performance of different classifiers (i.e., SVM, K-NN and LDA).

## 3. Results and Discussion

To evaluate the computer-aided early diagnosis of facial paralysis based on IRT, the following three key issues were discussed: (1) the difference of the facial temperature feature symmetry between facial paralysis patients and healthy population, (2) the difference of the texture feature symmetry between facial paralysis patients and healthy population, and the influence of different feature symmetry combinations on the early diagnosis of facial paralysis, especially whether the application of texture features contributes to the diagnosis of facial paralysis.

### 3.1. Symmetry Measurements of Temperature Features

**Figure 5:**
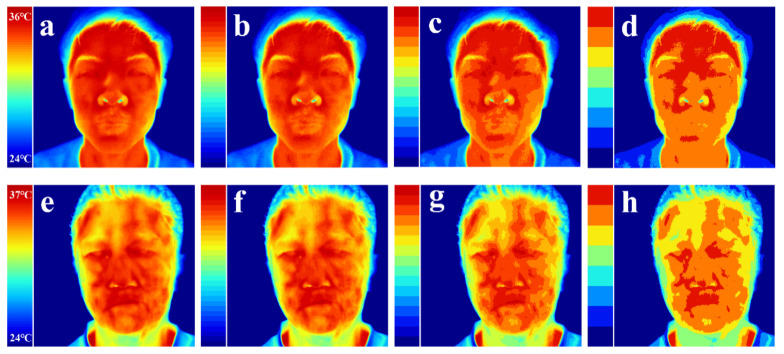
Comparison of facial thermal images between healthy subject and patient with facial paralysis using different pseudo-color methods. (**a**–**d**) are images of the healthy subject, while (**e**–**h**) are for the patient with facial paralysis. According to the given lower and upper temperature limits, the temperature values of all pixels in the thermal image are divided from low to high into 64 layers (**a**,**e**), 32 layers (**b**,**f**), 16 layers (**c**,**g**) and 8 layers (**d**,**h**), where each pixel is given a pseudo-color according to the level of its temperature value.

The degree of thermal symmetry on the left and right sides of the face can be observed intuitively through the pseudo-color of the facial temperature distribution. The facial thermal images of a healthy person and a patient with facial paralysis were compared using different pseudo-color methods presented in Figure 5. Due to the distribution of blood vessels on the human face, both patients with facial paralysis and normal people have the facial thermal characteristics of high temperature in the middle and low temperature on both sides, which is consistent with the research findings of Guan [47] and Liu et al. [14,36]. In addition, facial paralysis destroys the symmetry of temperature distribution between the left and right sides of the face. The quantitative evaluation is shown in Table 5.

**Figure 6:**
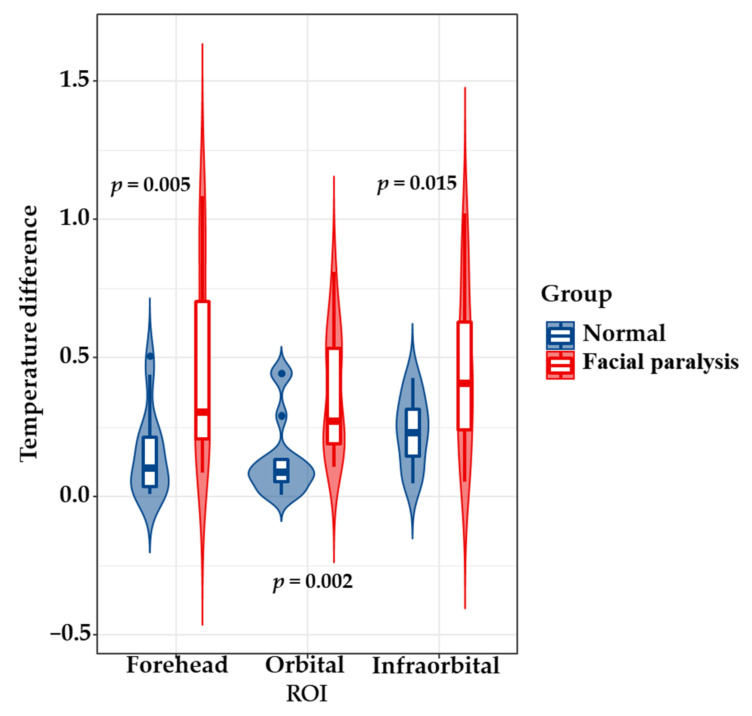
Comparison of *ΔT_mean_* between patients with facial paralysis and healthy people, where the horizontal line represents the median, the long box represents the inter-quartile range, and the outer contour curve represents the probability density of the data distribution.

**Figure 7:**
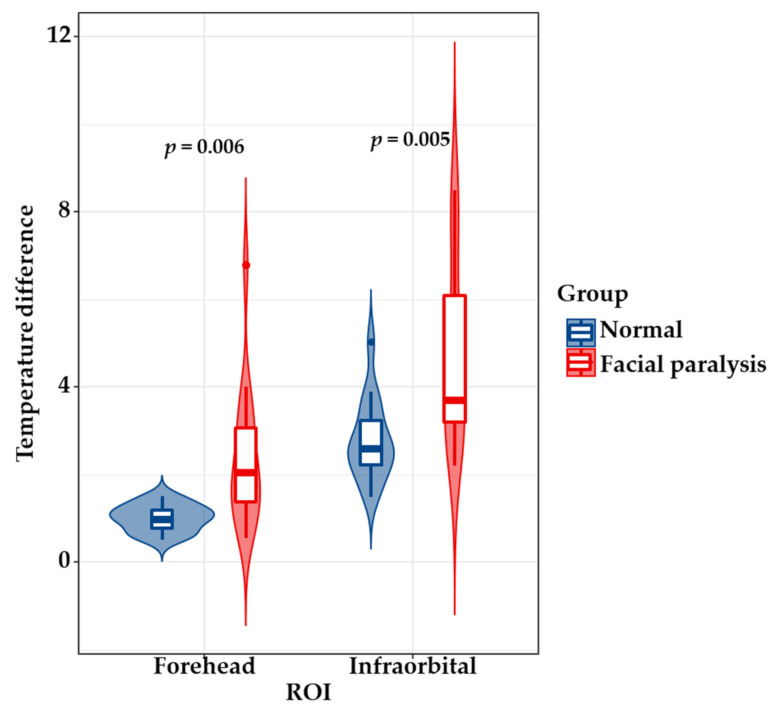
Comparison of *ΔT_max_* between patients with facial paralysis and healthy people, where the horizontal line represents the median, the long box represents the inter-quartile range, and the outer contour curve represents the probability density of the data distribution.

Temperature difference is one of the most commonly used methods to measure the symmetry of temperature features on both sides of the face [14]. Table 5 presents the statistics for the facial average temperature difference (Δ*T_mean_*) and maximum temperature difference (Δ*T_max_*) between facial paralysis patients and normal people. It can be seen from Table 5 that in the forehead, orbital, and infraorbital ROIs, the Δ*T_mean_* of facial paralysis patients were greater than that of the normal population (control group), and there were statistical differences between the two groups (Figure 6). Additionally, the Δ*T_max_* of patients with facial paralysis in the forehead and infraorbital ROIs were greater than that of the normal population, and there were statistical differences between the two groups (Figure 7). These results show that the symmetry of temperature features measured by Δ*T_mean_* and Δ*T_max_* can preliminarily distinguish patients with facial paralysis from normal people, and the symmetry of temperature features with statistical differences can be used as the selected features for early diagnosis of facial paralysis.

Drawing comparison to previous studies [14], the results of Δ*T_mean_* are seen to be consistent, but the results of Δ*T_max_* are not completely identical. The reasons for the difference are (1) the calculation method of Δ*T_max_* is different, i.e., the previous study [14] only considered the difference of the maximum temperature between the two ROIs, but this study highlights the difference between the temperature features of the two ROIs through combining the maximum and minimum temperature; (2) the size of ROIs, especially in the orbital region, was not entirely consistent; and (3) the sample size was enlarged in this study.

Figure 6 and Figure 7 further show the difference in temperature feature symmetry between patients with facial paralysis and healthy people. According to the interquartile ranges and the probability density of data distribution, it can be found that the inter-subject variability of patients with facial paralysis was greater than that of normal people; this is mainly caused by the inconsistent severity of symptoms in different parts of different facial paralysis patients. In addition, Δ*T_mean_* and Δ*T_max_* cannot be used in the diagnosis of facial paralysis in nasal and mouth regions, mainly due to the large variation of temperature features in these regions in the normal population.

### 3.2. Symmetry Measurements of Texture Features

Through the observation of the subjects’ facial thermograms, the symmetry of the facial temperature distribution on the ipsilateral and contralateral sides of patients with facial paralysis was lower than that of the normal population. The current studies [14] only analyzed the temperature features, but ignored the temperature spatial distribution, which may result in overlooking the corresponding features that are valuable for early diagnosis of facial paralysis. The texture features can effectively represent the temperature spatial distribution on the thermal image. Therefore, the classical texture representation method of GLCM was selected to calculate the temperature spatial distribution in this study.

In this study, the Manhattan and Euclidean distances were used to evaluate the symmetry of texture features on the left and right sides of the face. As shown above, there were 32 texture feature symmetries between each pair of ROIs on the left and right sides of the infrared thermal image; half of them used Manhattan distance and the other half used Euclidean distance. Table 6 and Table 7 summarize the statistical results of texture feature symmetry using Manhattan and Euclidean distances respectively, and only the feature symmetry with statistical differences between facial paralysis patients and healthy people were included.

From Table 6 and Table 7, the following conclusions are drawn: (1) there are significant differences in the spatial distribution of facial temperature on the left and right sides between patients with facial paralysis and healthy people; (2) in comparison with temperature features, texture features can be used for the diagnosis of facial paralysis in all facial ROIs, including nasal and mouth regions; (3) among all texture features, energy and homogeneity are the most valuable diagnostically; (4) the inter-subject variability of patients with facial paralysis was greater than that of the normal population.

### 3.3. Performance Measurement of Different Feature Symmetry Combinations for Diagnosis of Facial Paralysis

Different feature symmetry combinations have great influence for the diagnostic performance of classifiers. In this study, an easy-to-use feature symmetry selection method was used, i.e., we selected the temperature and texture feature symmetry with statistical differences between patients with facial paralysis and normal population. Therefore, the performance of different feature symmetry combinations was compared: (1) all temperature feature symmetries and the temperature feature symmetries with statistical differences (Table 8), (2) all texture feature symmetries and the texture feature symmetries with statistical differences (Table 9), (3) the combinations of all temperature and texture feature symmetries and the combinations of the feature symmetries with statistical differences (Table 10).

The simplest feature symmetry selection method involves inputting all temperature and texture feature symmetries. When only temperature feature symmetry was selected, the accuracy of SVM in the diagnosis of facial paralysis was 0.833 (Table 8). When only texture feature symmetry was input, the diagnostic accuracy of facial paralysis was 0.767 (Table 9). When all temperature and texture feature symmetries were combined, the diagnostic accuracy of facial paralysis was 0.8 (Table 10). With the increase of feature dimensionality and use of texture features, there was no improvement in the classification performance after using only temperature feature symmetry. This attributed to the lack of feature selection. More features are not necessarily better, but features that are more complementary and differentiated should be selected.

For the results in Table 5, Table 6 and Table 7, t-test was used to select temperature and texture feature symmetries with significant differences between the two groups of subjects from all features. When only temperature feature symmetry with significant differences was used, the diagnostic accuracy of facial paralysis was 0.767 (Table 8). When only texture feature symmetry with significant differences was used, the accuracy was 0.833 (Table 9). After combining the above two feature symmetries, the diagnostic accuracy of facial paralysis was 0.933 (Table 10). In conclusion, texture feature symmetry is helpful for diagnosis of facial paralysis, and the combination of temperature and texture feature symmetry with significant differences can improve the diagnostic accuracy of facial paralysis compared to using a single type of feature symmetry.

**Figure 8:**
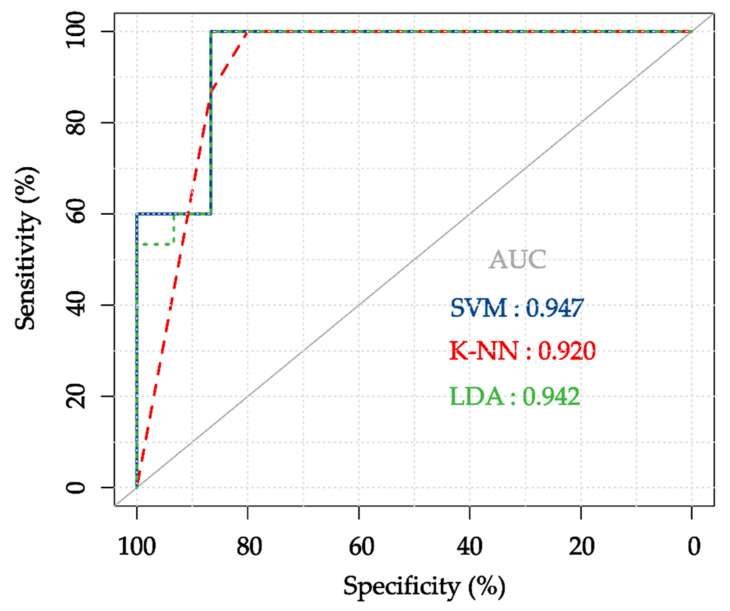
Diagnostic performance comparison of SVM, k-NN and LDA for facial paralysis.

Moreover, SVM was selected as a diagnostic classifier for facial paralysis. The AUC was used for comparing the generalization performance of SVM with other two typical classifiers, as shown in Figure 8. SVM has good adaptability to the diagnosis of facial paralysis, and the AUC is 0.947, i.e., higher than k-NN and LDA. In addition, this study proposed an SVM diagnosis system for facial paralysis based on temperature and texture features, which have higher sensitivity, specificity, and AUC than those of previous similar studies [14,34] (Table 11). The reasons for these results are: (1) SVM is good at small sample machine learning; (2) the previous studies only used single type features and threshold method; (3) two types of complementary thermal features were combined, and supervised learning was used to train the classification parameters in this study.

HB score is a conventional method to evaluate facial paralysis. Based on the asymmetry of facial expressions of the subjects, the facial nerve function is evaluated as normal (grade I) and facial paralysis (grade II-VI) by physicians. The drawback is that it relies on a subjective judgment with significant inter-rater variation. In order to solve the problem, the computer-aided diagnosis methods for facial paralysis based on HB Score are developed, and the highest accuracy of distinguishing facial paralysis from normal is 0.923 [1,9,48]. In this study, the accuracy of the proposed method based on temperature asymmetry is 0.933. We consider this accuracy excellent because relevant studies have shown that the reliability of HB Score itself ranges from 0.8 to 0.93 [49]. Because the data set used for training the classifier is calibrated by the conventional method, the accuracy has reached the upper limit of the conventional method. In addition to the comparison with the above literatures [1,9,48,49], the proposed method and the conventional method will be used to diagnose the same patients for a clearer comparison in the future study. The proposed method verifies the feasibility of infrared thermal imaging in the diagnosis of facial paralysis, which is beneficial compared with traditional methods. This is mainly caused by two reasons: (1) the automatic thermal asymmetry analysis algorithm can avoid the inter-rater variation, (2) subjects do not need to make a series of facial expressions and it leads to higher coordination.

The patients with very mild symptoms may be missed diagnosed through HB scores. In the proposed thermal imaging diagnosis, no patients with facial paralysis was missed diagnosed, but 13.3% of healthy subjects were misdiagnosed as facial paralysis due to the variation of the facial temperature asymmetry. This method is a useful supplement to the conventional method. The asymmetry of facial temperature distribution in patients with facial paralysis is not completely consistent with the HB scores. Patients with mild symptoms may have significant facial thermal asymmetry, which needs to be verified by expanding the data set in future studies.

In order to assist physicians in clinical decision-making, our final goal is to develop a facial paralysis evaluation system based on infrared thermal imaging. This process is divided into four steps. Firstly, the facial temperature distribution between patients with facial paralysis and healthy people is compared to find out the feature symmetry combination with significant difference. Secondly, an automatic method which distinguishes between facial paralysis and normal is developed using thermal asymmetry analysis. Thirdly, the differences in thermal images between facial paralysis and other diseases that change the facial temperature distribution are analyzed, and a thermal image analysis method to distinguish facial paralysis from other diseases will be further explored. Finally, the correlation between thermal asymmetry and HB scores is analyzed, and a computerized facial paralysis grading system is explored based on thermal asymmetry. This study focuses on the first two steps. In order to complete the distinction between facial paralysis and normal on thermal images, the exclusion criteria are used to eliminate the interference of certain diseases on the diagnosis of facial paralysis. The exclusion criteria can enhance the sensitivity and specificity of thermal asymmetry analysis and simplify the complexity of this research.

Certain diseases may have similar facial temperature manifestations, such as facial paralysis, stroke, Parkinson, temporomandibular arthritis, etc. These diseases may lead to the facial temperature asymmetry, but the degree and site of temperature asymmetry may be different. However, this study only analyzes the difference in facial thermal images between facial paralysis and healthy people. In addition, a general framework for analyzing facial thermal asymmetry is proposed in this study. In the future research, it is necessary to distinguish the diseases with similar temperature features by changing the facial ROIs.

The automatic output results of the developed computer-aided thermal image analysis system are divided into two categories: facial paralysis and normal. In future studies, the output of the extended system will be the severity of facial paralysis (six classes, normal to complete paralysis [48,49]). Accordingly, the following methods could be adopted: (1) enlarging the sample dataset and increasing the number of subjects with different facial paralysis severity; (2) analyzing the correlation between temperature and texture features and the severity of facial paralysis; (3) evaluating other temperature and texture features, such as Histogram of oriented gradients and Gabor filters; (4) extending the application to the curative effect evaluation of facial paralysis.

## 4. Conclusions

In this study, a computer-aided thermal image analysis method for early diagnosis of facial paralysis was proposed. The facial ROIs were automatically segmented using corner and edge detection, which improves the reliability of thermal image analysis. After measuring the temperature and texture feature symmetries of the bilateral ROIs of the subject’s face, it was found that there was a significant difference in the symmetry of facial temperature distribution between patients with facial paralysis and normal population. The SVM was used to evaluate the degree of symmetry between thermal features, and its sensitivity, specificity, and AUC in the diagnosis of facial paralysis were superior compared to existing studies. In conclusion, the combination of temperature and texture features can effectively describe the facial temperature distribution of patients with facial paralysis, and the automatic diagnosis method of facial paralysis based on IRT is feasible. In future work, the computer-aided thermal asymmetry analysis could be used to evaluate the severity of facial paralysis.

## Figures and Tables

**Table 1:** Distribution of the House-Brackmann scores in the subjects.

Subject	Score	Description	Number of Subjects	
Healthy individuals	I	Normal	45	
Patients with facial paralysis	II	Slight	3	Total 45
	III	Moderate	8	
	IV	Moderately severe	14	
	V	Severe	20	
	VI	Total paralysis	0	

**Table 2:** Automatic segmentation algorithm of facial ROIs.

1: Input grayscale image ← Original thermal image preprocessing
2: Background segmentation ← Otsu method
3: Corner detection ← Harris operator and morphological operations
4: Key point positioning of nostril and pupil ← Gray projections
5: Edge Detection ← Canny edge detector and morphological operations
6: Key point positioning of the mouth ← Gray projections
7: Key point positioning of canthus and eyebrow ← Fusion of corner and edge features
8: ROI segmentation ← Combining facial key points and anthropometry

**Table 3:** Four second-order texture features calculated by GLCM.

Feature	Description	Mathematical Expression
Contrast	Sharpness and depth of thermal texture	$\sum_{i}\sum_{j}{\left|i-j\right|}^{2}P\left(i,j\right)$
Correlation	Correlation of local temperature distribution	$\sum_{i}\sum_{j}\frac{\left(ij\right)P\left(i,j\right)-\mu_{x}\mu_{y}}{\sigma_{x}\sigma_{y}}$
Energy	Temperature distribution uniformity and fineness	$\sum_{i}\sum_{j}P{\left(i,j\right)}^{2}$
Homogeneity	Homogeneity of thermal texture and local variation of temperature distribution	$\sum_{i}\sum_{j}\frac{P\left(i,j\right)}{1+{\left(i-j\right)}^{2}}$

**Table 4:** Confusion Matrix of Diagnosis Results.

Actual Class	Predicted Class	
	Facial Paralysis	Normal
Facial paralysis	TP	FN
Normal	FP	TN

**Table 5:** Symmetry measurements of facial temperature features between patients with facial paralysis and healthy population.

ROI	Features	ΔT of ROIs (Mean ± SD)		
		Control Group	Facial Paralysis Group	*p* Value
Forehead region	*ΔT_mean_*	0.15 ± 0.14	0.47 ± 0.34	0.005 **
	*ΔT_max_*	1.01 ± 0.31	2.35 ± 1.59	0.006 **
Orbital region	*ΔT_mean_*	0.14 ± 0.13	0.38 ± 0.21	0.002 **
	*ΔT_max_*	3.81 ± 1.36	3.64 ± 1.56	0.305
Infraorbital region	*ΔT_mean_*	0.24 ± 0.12	0.46 ± 0.29	0.015 *
	*ΔT_max_*	2.78 ± 0.92	4.72 ± 2.16	0.005 **
Nasal Region	*ΔT_mean_*	0.42 ± 0.27	0.47 ± 0.36	0.668
	*ΔT_max_*	5.40 ± 1.55	4.67 ± 2.23	0.311
Mouth region	*ΔT_mean_*	0.35 ± 0.20	0.39 ± 0.28	0.602
	*ΔT_max_*	3.67 ± 1.44	4.04 ± 2.19	0.585

* *p* < 0.05, ** *p* < 0.01.

**Table 6:** Symmetry measurements of texture features using the Manhattan distance (the offset distance Δ of the GLCM is set to 2 and 5, and the maximum gray level is set to 16 and 32).

ROI	Features	Feature Symmetry Value (Mean ± SD)		
		Control Group	Facial Paralysis Group	*p* Value
Forehead region	Energy (5,16)	0.018 ± 0.017	0.04 ± 0.027	0.015 *
	Energy (2,32)	0.013 ± 0.01	0.035 ± 0.036	0.035 *
	Energy (5,32)	0.012 ± 0.009	0.033 ± 0.032	0.025 *
Orbital region	Contrast (5,16)	6.402 ± 8.948	14.718 ± 12.337	0.050 *
	Contrast (5,32)	25.316 ± 34.117	59.547 ± 48.876	0.042 *
Infraorbital region	Homogeneity (2,16)	0.035 ± 0.017	0.061 ± 0.044	0.043 *
	Homogeneity (5,16)	0.076 ± 0.039	0.134 ± 0.085	0.028 *
Nasal Region	Energy (2,16)	0.126 ± 0.101	0.439 ± 0.447	0.017 *
	Energy (5,16)	0.123 ± 0.077	0.435 ± 0.477	0.022 *
	Energy (2,32)	0.054 ± 0.039	0.176 ± 0.162	0.011 *
	Energy (5,32)	0.049 ± 0.035	0.152 ± 0.133	0.009 **
Mouth region	Homogeneity (2,16)	0.05 ± 0.018	0.085 ± 0.037	0.004 *
	Energy (2,32)	0.079 ± 0.08	0.236 ± 0.221	0.019 *
	Energy (5,32)	0.072 ± 0.077	0.21 ± 0.218	0.034 *

* *p* < 0.05, ** *p* < 0.01.

**Table 7:** Symmetry measurements of texture features using the Euclidean distance (the offset distance Δ of the GLCM is set to 2 and 5, and the maximum gray level is set to 16 and 32).

ROI	Features	Feature Symmetry Value (Mean ± SD)		
		Control Group	Facial Paralysis Group	*p* Value
Forehead region	Energy (2,16)	0.012 ± 0.011	0.022 ± 0.016	0.048 *
	Energy (5,16)	0.01 ± 0.008	0.021 ± 0.014	0.013 *
	Energy (2,32)	0.007 ± 0.005	0.018 ± 0.019	0.031 *
	Energy (5,32)	0.006 ± 0.005	0.017 ± 0.017	0.021 *
Orbital region	Homogeneity (2,16)	0.046 ± 0.032	0.086 ± 0.061	0.032 *
	Contrast (5,16)	3.631 ± 5.059	9.402 ± 8.338	0.031 *
	Homogeneity (5,16)	0.081 ± 0.05	0.133 ± 0.068	0.025 *
	Homogeneity (2,32)	0.059 ± 0.041	0.097 ± 0.046	0.022 *
	Contrast (5,32)	14.423 ± 19.502	37.935 ± 33.293	0.027 *
Infraorbital region	Homogeneity (2,16)	0.02 ± 0.01	0.035 ± 0.024	0.044 *
	Homogeneity (5,16)	0.046 ± 0.025	0.077 ± 0.048	0.038 *
Nasal Region	Energy (2,16)	0.066 ± 0.051	0.221 ± 0.231	0.017 *
	Energy (5,16)	0.068 ± 0.041	0.22 ± 0.247	0.025 *
	Energy (2,32)	0.029 ± 0.02	0.09 ± 0.083	0.009 **
	Energy (5,32)	0.027 ± 0.018	0.079 ± 0.068	0.008 **
Mouth region	Homogeneity (2,16)	0.03 ± 0.011	0.048 ± 0.021	0.009 **
	Energy (2,32)	0.041 ± 0.042	0.12 ± 0.113	0.017 *
	Energy (5,32)	0.039 ± 0.042	0.107 ± 0.112	0.036 *

* *p* < 0.05, ** *p* < 0.01.

**Table 8:** Diagnostic performance of facial paralysis using different temperature feature symmetry combinations.

Feature Symmetry	Dimensions	Accuracy	Sensitivity	Specificity	Precision	F-Score
All Δ*T_mean_*	5	0.7	1	0.4	0.625	0.489
Δ*T_mean_* with significant differences	3	0.833	0.8	0.867	0.857	0.862
All Δ*T_max_*	5	0.833	0.8	0.867	0.857	0.862
Δ*T_max_* with significant differences	2	0.8	0.933	0.667	0.737	0.700
ΔT1: All Δ*T_mean_* and Δ*T_max_*	10	0.833	0.8	0.867	0.857	0.862
ΔT2: Δ*T_mean_* and Δ*T_max_* with significant differences	5	0.767	0.867	0.667	0.722	0.693

**Table 9:** Diagnostic performance of facial paralysis using different texture feature symmetry combinations.

Feature Symmetry	Dimensions	Accuracy	Sensitivity	Specificity	Precision	F-Score
F1: Texture symmetries by Manhattan distance	80	0.767	0.867	0.667	0.722	0.693
F2: Texture symmetries by Euclidean distance	80	0.767	0.8	0.733	0.75	0.742
F3: F1 with significant differences	14	0.8	0.8	0.8	0.8	0.8
F4: F2 with significant differences	18	0.767	0.8	0.733	0.75	0.742
F1 + F2	160	0.633	0.467	0.800	0.700	0.747
F3 + F4	32	0.833	0.8	0.867	0.857	0.862

**Table 10:** Diagnostic performance of facial paralysis using a combination of temperature and texture feature symmetry.

Feature Symmetry	Dimensions	Accuracy	Sensitivity	Specificity	Precision	F-Score
ΔT1 + F1 + F2	170	0.8	0.867	0.733	0.765	0.749
ΔT1 + F3 + F4	42	0.9	1	0.8	0.833	0.816
ΔT2 + F3 + F4	37	0.933	1	0.867	0.882	0.874

**Table 11:** Comparison between proposed method and existing studies.

Study	Features Used	Classifier	Sensitivity	Specificity	AUC
[34]	Texture (LBP)	Threshold	0.860	0.890	-
[14]	Temperature	Threshold	0.867	0.800	0.818
Proposed study	Composite features	SVM	1	0.867	0.947

## Data Availability

The datasets generated and analyzed during the current study are available from the corresponding authors on reasonable request.
